# Femoral Neck Nonunion Associated With Delayed Union of Ipsilateral Femoral Shaft Fracture

**DOI:** 10.7759/cureus.15612

**Published:** 2021-06-12

**Authors:** John A Santoshi, Lingaraj Reddy, Udit Agrawal

**Affiliations:** 1 Orthopaedics, All India Institute of Medical Sciences, Bhopal, IND; 2 Orthopaedics, Pondicherry Institute of Medical Sciences, Puducherry, IND

**Keywords:** concomitant hip and femoral shaft fracture, ipsilateral femoral neck shaft, nonunion, intertrochanteric osteotomy, pauwels' osteotomy, retrograde nailing, delayed union

## Abstract

We report the case of a 36-year-old man, who presented to us five months after the initial trauma. He had been treated elsewhere with a cephalomedullary femoral nail. He described severe pain in his right thigh and groin that confined him to a wheelchair. He had shortening of the right lower limb and painful restriction of movements of the right hip. Radiographs demonstrated hypertrophic callus with a gap at the femoral shaft while the neck fracture was in varus malalignment with bone resorption; the neck fracture been fixed using two hip screws that were missing the nail. The patient was managed with removal of the previous hardware, reamed retrograde nailing and Pauwels’ intertrochanteric valgus osteotomy fixed using a 120^o^ double-angled condylar blade plate. Both the fracture sites were not opened. Postoperatively, the femoral shaft showed radiographic evidence of union at three months, while the femoral neck and the intertrochanteric osteotomy site had united at five months. As per the Friedman and Wyman criteria, our patient has a “good” outcome at the four-year follow-up.

## Introduction

Concomitant ipsilateral femoral shaft and neck fractures usually result from high-velocity injuries in young patients [1-3]. The incidence of these injuries is approximately 1% to 9% of all femoral shaft fractures [4,5]. In polytraumatized patients, these injuries are often missed and delayed diagnoses are common as the femoral neck fracture pattern is either undisplaced or minimally displaced. Longitudinal compression force is responsible for this fracture pattern; the femoral shaft absorbs much of the energy before causing a vertical, minimally displaced, and easily masked femoral neck fracture [6]. The reported incidence of delayed diagnosis of these injuries ranges from 19% to 50% of patients during the initial examination [4]. Factors attributed to the missed or delayed diagnoses include the presence of accompanying distracting injuries, inadequate pre- and intra-operative imaging of the injuries, and the undisplaced or minimally displaced femoral neck fracture pattern [4,5]. This delay in diagnosis and treatment predisposes to complications like nonunion and avascular necrosis [4].

While the incidence of ipsilateral femoral neck and shaft fractures that go on to nonunion is not known the likelihood of these injuries to go on to nonunion is higher than isolated femoral neck or femoral shaft fractures. The possible explanations for this are, the injury is more likely to be a consequence of high energy trauma resulting in increased vascular disruption, and secondly, the free segment between the two fracture sites may create more motion at the proximal and distal fracture sites than would be encountered at an isolated femur fracture site. Other factors like significant soft tissue disruption, use of unreamed small-diameter intramedullary nails for the shaft fracture, prolonged delay in weight-bearing, inadequate fracture stability, and smoking may also contribute to the progression to nonunion [3].

Concomitant ipsilateral femoral neck and shaft nonunions may present difficult diagnostic and treatment challenges. These patients frequently complain of pain and one could be dealing with referred pain from one nonunion site masking the pain from the second nonunion site. Standard radiographic imaging has its limitations due to the presence of hardware making the determination of union difficult. Furthermore, treatment is complicated by the previous hardware and the difficulty in selecting optimal fixation methods secondary to the lack of literature on the topic [3].

We report a case of femoral neck nonunion associated with delayed union of the ipsilateral femoral shaft which was successfully treated using valgus intertrochanteric osteotomy for the femoral neck and retrograde nail for the femoral shaft.

The patient was informed that data concerning the case would be submitted for publication, and he provided consent.

## Case presentation

**Figure 1:**
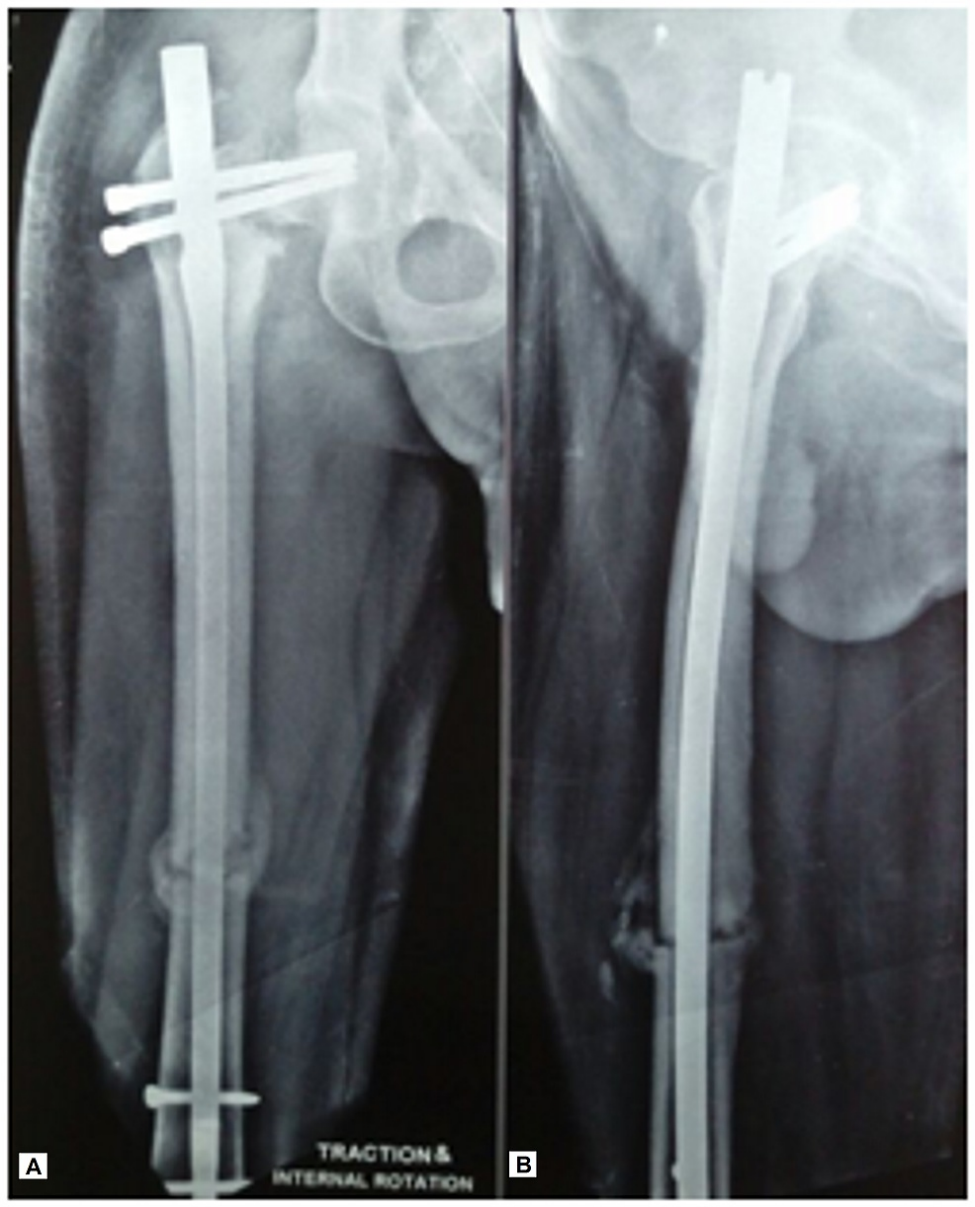
Radiographs of (A) the right hip and (B) thigh showing atrophic nonunion of the femoral neck with resorption of bone and varus malalignment along with hypertrophic callus with gap at the ipsilateral femoral shaft, on presentation.

A 36-year-old man presented to the orthopedic outpatient clinic with complaints of pain and inability to bear weight on the right lower limb following a road traffic accident five months back. He had sustained an injury to the right lower limb and had been treated elsewhere with a cephalomedullary femoral nail. Details of previous treatment including the pre-treatment radiographs were not available. He had never borne weight on the right lower limb following the primary surgery. He described severe pain in his right thigh and groin that confined him to a wheelchair. He had shortening of the right lower limb and painful restriction of movements of the right hip. Radiographs showed delayed union at the femoral shaft fracture site while the femoral neck was in varus malalignment with bone resorption. The neck fracture had been fixed using two hip screws that were missing the nail; the Pauwels' angle measured ~65° (Figures 1A, 1B). Workup for infection was negative and he was neither a diabetic nor a smoker.

**Figure 2:**
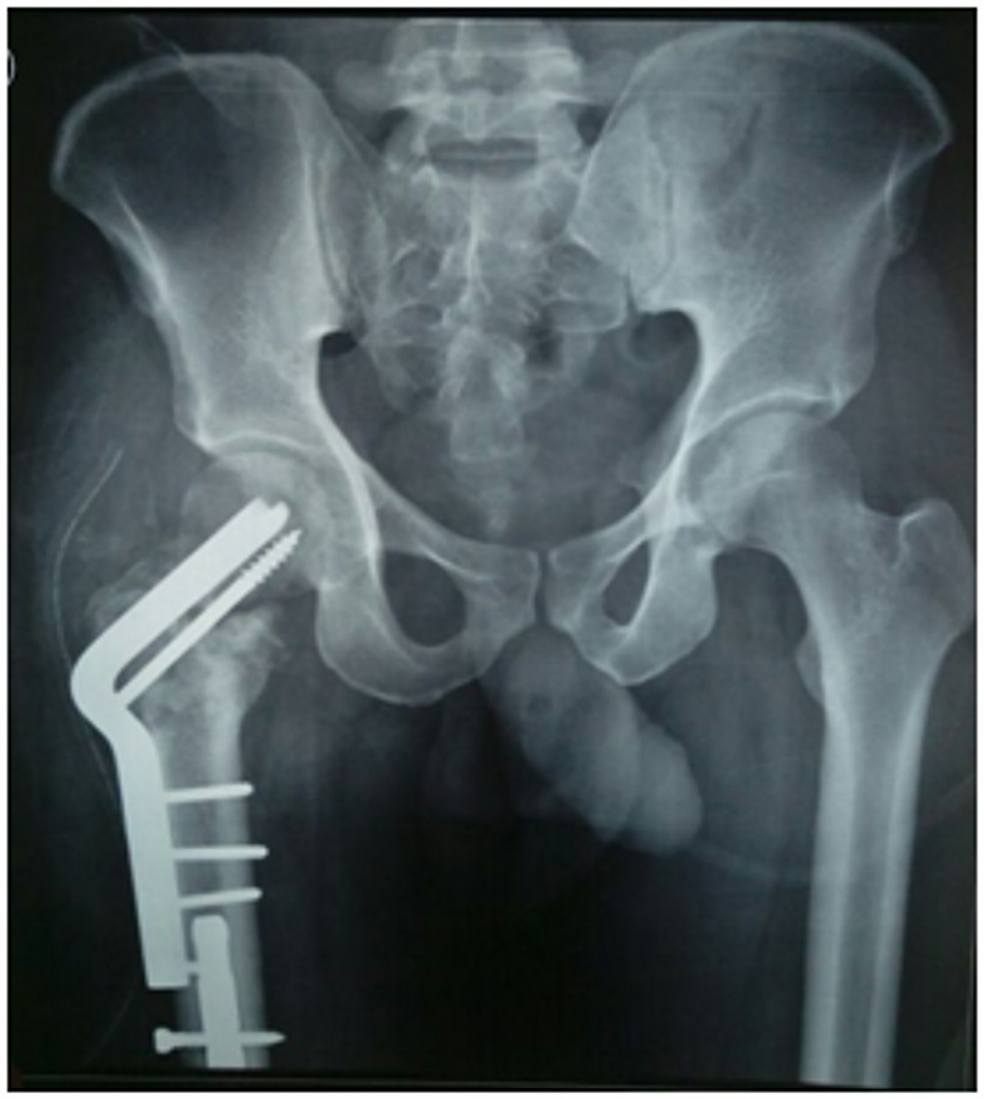
Immediate postoperative radiograph showing fixation of the Pauwels' intertrochanteric osteotomy with angled blade plate

**Figure 3:**
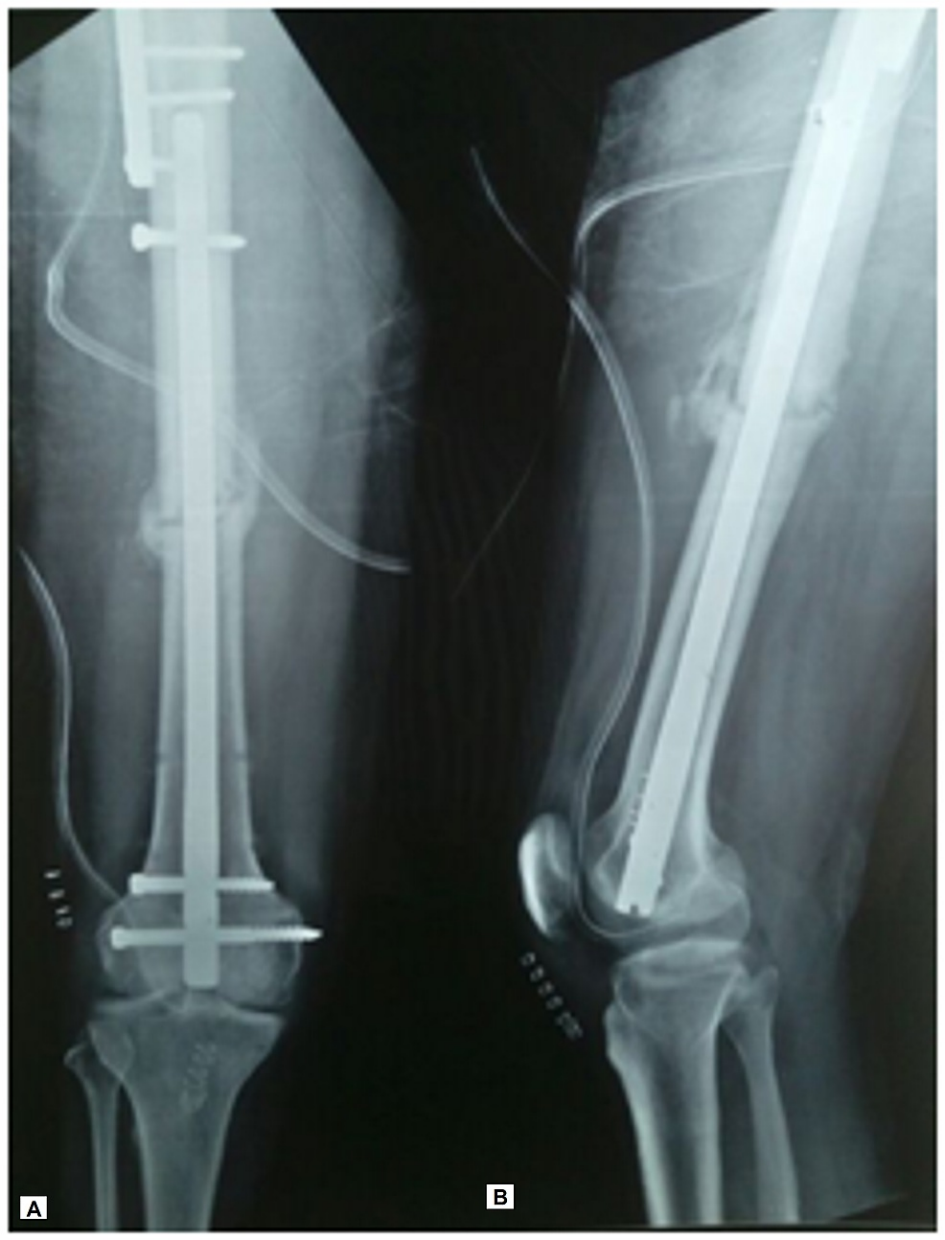
(A, B) Immediate postoperative radiograph showing fixation of femoral shaft using reamed retrograde nail

At revision surgery, the previous hardware was removed and a reamed retrograde femoral nail was inserted and locked in compression; the shaft fracture site was not opened. Next, to correct the varus neck malalignment, Pauwels’ intertrochanteric osteotomy was performed. A 30° wedge of bone was removed from the lateral cortex, and a 120° double-angled condylar blade plate was inserted under fluoroscopic imaging; the femoral neck nonunion was not opened. At the site of overlap of the implants in the subtrochanteric region, we could pass a unicortical screw from the distal hole of the blade plate into the proximal interlocking hole of the nail though it could not be passed through and through; this served to avoid potential stress riser at the subtrochanteric region (Figures 2 and 3A, 3B). Postoperatively, he was placed on foot-flat weight-bearing status for six weeks with a progressive increase in weight-bearing thereafter.

**Figure 4:**
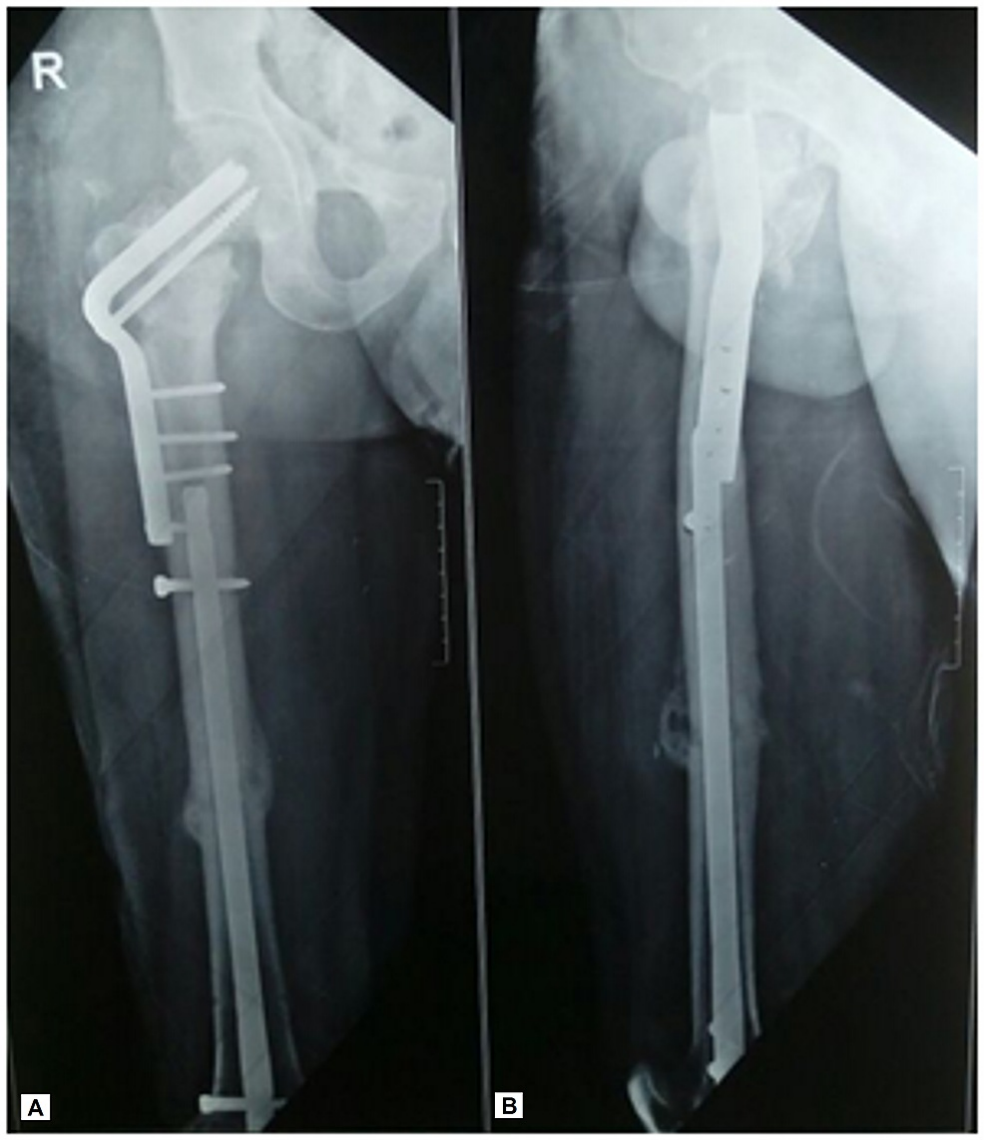
(A, B) Radiographs taken three months postoperatively showing union at the femoral shaft

**Figure 5:**
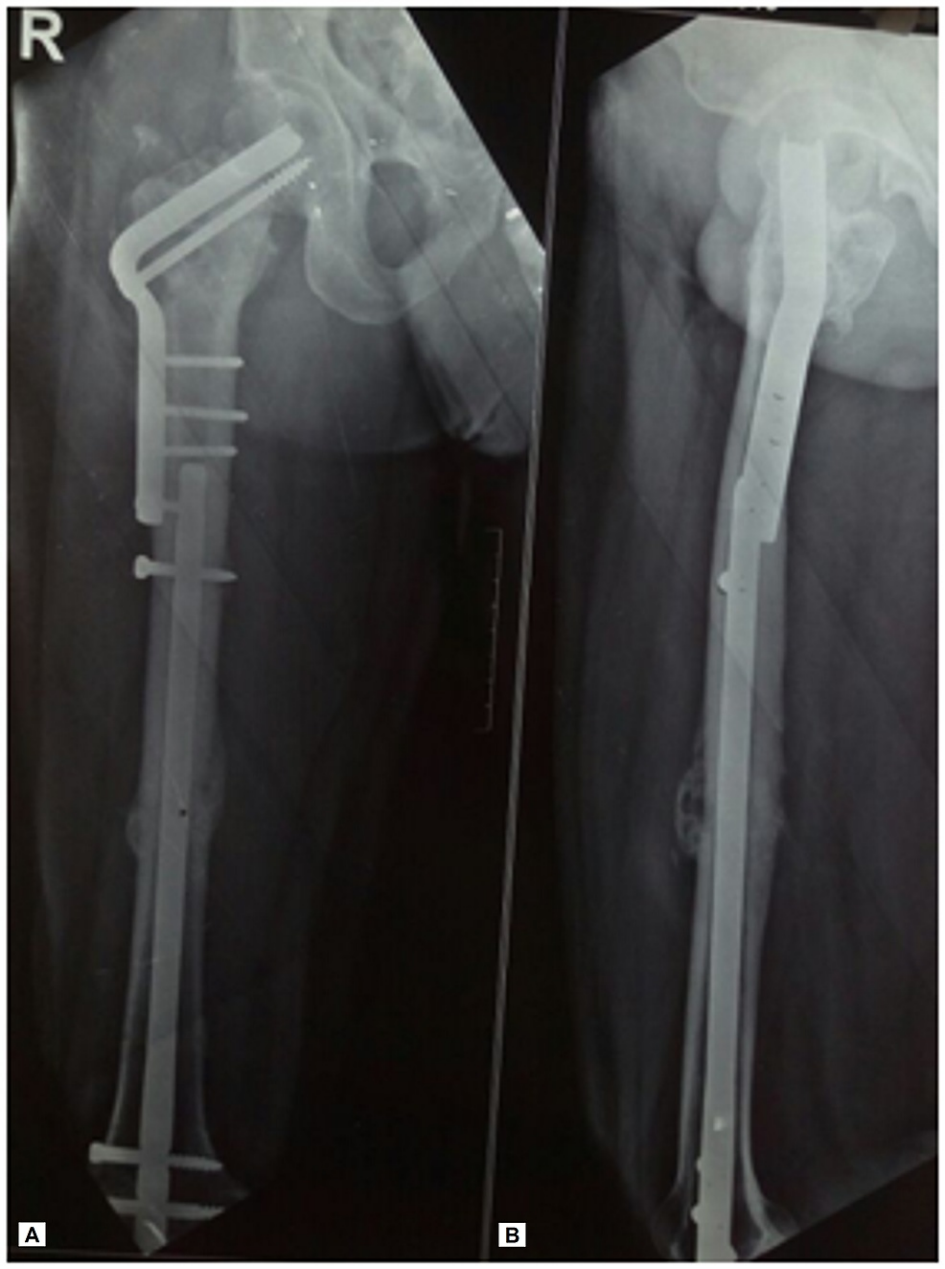
(A, B) Radiographs taken five months postoperatively showing union at the femoral neck and osteotomy site

At three months postoperatively, the femoral shaft showed radiographic evidence of union (Figures 4A, 4B) while the femoral neck and the intertrochanteric osteotomy site showed union at five months postoperatively (Figures 5A, 5B).

**Figure 6:**
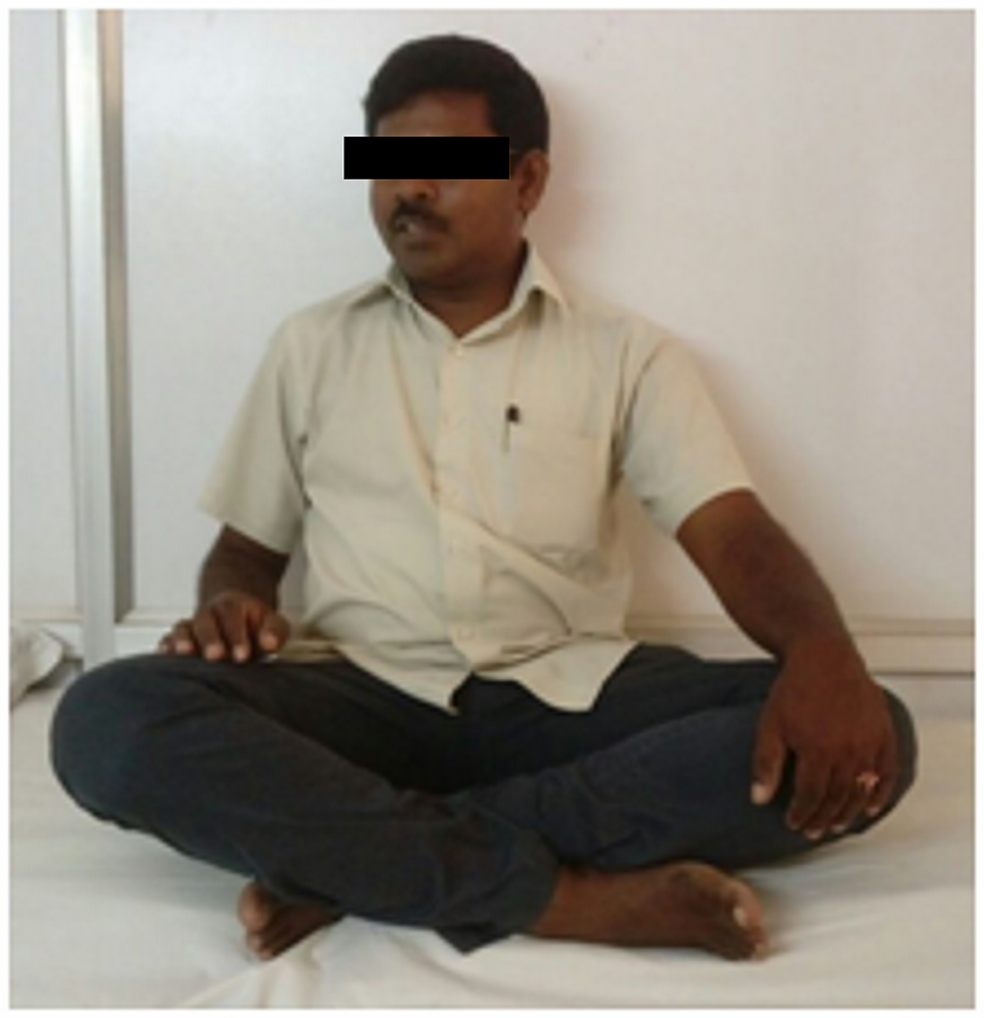
Clinical photograph at four-year follow-up showing the patient sitting cross-legged comfortably

At four-year follow-up, he had no complaints and was functioning well with regard to activities of daily living (ADL). He was able to sit cross-legged, squat and stand on the affected leg (Figure 6).

He had a minimal limb length discrepancy that measured around 1 cm but the patient was not bothered much about it. According to the Friedman and Wyman Criteria [7], our patient's functional outcome was found to be “good” with no limitation of ADL, no pain and <20% loss of hip or knee motion.

## Discussion

Nonunion of the femoral neck with bone resorption and varus malalignment along with the presence of implant in the femoral head and delayed union of the ipsilateral shaft fracture made this a challenging case both from the surgical point of view as well as fracture healing perspective. When dealing with fresh concomitant ipsilateral femoral neck and shaft fractures, there is consensus that these injuries should be managed operatively and for which as many as 30 different fracture fixation strategies have been described. These are often classified by the number of devices used, as single or double device fixation, depending on whether a single device has been for fixation of both fractures, or separate devices have been used to fix each fracture [5].

While the incidence of ipsilateral femoral neck and shaft fractures that go on to nonunion is not known, when dealing with concomitant ipsilateral femoral neck and shaft nonunion, there is very little literature to guide treatment. In the absence of controlled studies, the best method for managing these combined fractures continues to be supported by subjective reasoning [3]. Modalities, like valgus osteotomy, vascularized bone graft, internal fixation with or without bone grafting, and replacement arthroplasty, have been described for the treatment of femoral neck nonunion [8-11]. We chose exchange nailing as it is an accepted modality for femoral shaft delayed union [12].

Alfonso et al. [3] reported on the successful treatment of three cases with concomitant ipsilateral femoral neck and shaft nonunions seen six to seven months after the initial trauma. One patient was treated with cephalomedullary spiral blade while the other two were treated with Pauwels’ osteotomy and retrograde nail. They opened the fracture sites in two patients for placing cancellous bone graft and bone stimulator.

Labza et al. [4] reported a case of polytrauma treated on the day of admission with closed reduction and external fixation of the femoral shaft fracture. Ipsilateral femoral neck fracture had been missed on the initial computed tomography (3 mm thickness) of the pelvis. The patient was treated on post-injury day three with a cephalomedullary nail. The femoral shaft united but the neck progressed to nonunion with lag-screw cut-out. This was treated with bipolar arthroplasty one year later.

Muller et al. [13] reported a case in which the femoral shaft fracture was stabilized with an intramedullary nail. Postoperative radiographs showed a missed ipsilateral femoral neck fracture which was subsequently stabilized with two screws using the miss-a-nail technique. Six months later, he presented with nonunion of the femoral neck and delayed union of the femoral shaft, which was treated with valgus osteotomy for the femoral neck and plating for the shaft. Both the sites had united at sixth-month postoperative follow up. 

Chan et al. [14] reported a case of segmental fracture femur treated with an interlocking nail. Ipsilateral femoral neck fracture was noted on the postoperative radiographs. Six months later the patient presented with femoral neck nonunion along with the delayed union of the femoral shaft. He was treated by valgus osteotomy and vascularized fibula bone grafting for the neck and retrograde nail for the shaft. They achieved radiological union at the fourth-month follow-up.

The primary osteosynthesis performed in our patient failed as the cephalomedullary nail had not been interlocked proximally as the hip screws were found to be missing the nail. This was probably due to a mismatch between the jig and the nail. Such intraoperative complications due to manufacturing or implant assembly related problems have been reported earlier [15]. The implants failed to provide axial and rotational stability at both the fracture sites in our patient, as shown in Figure 1.

**Figure 7:**
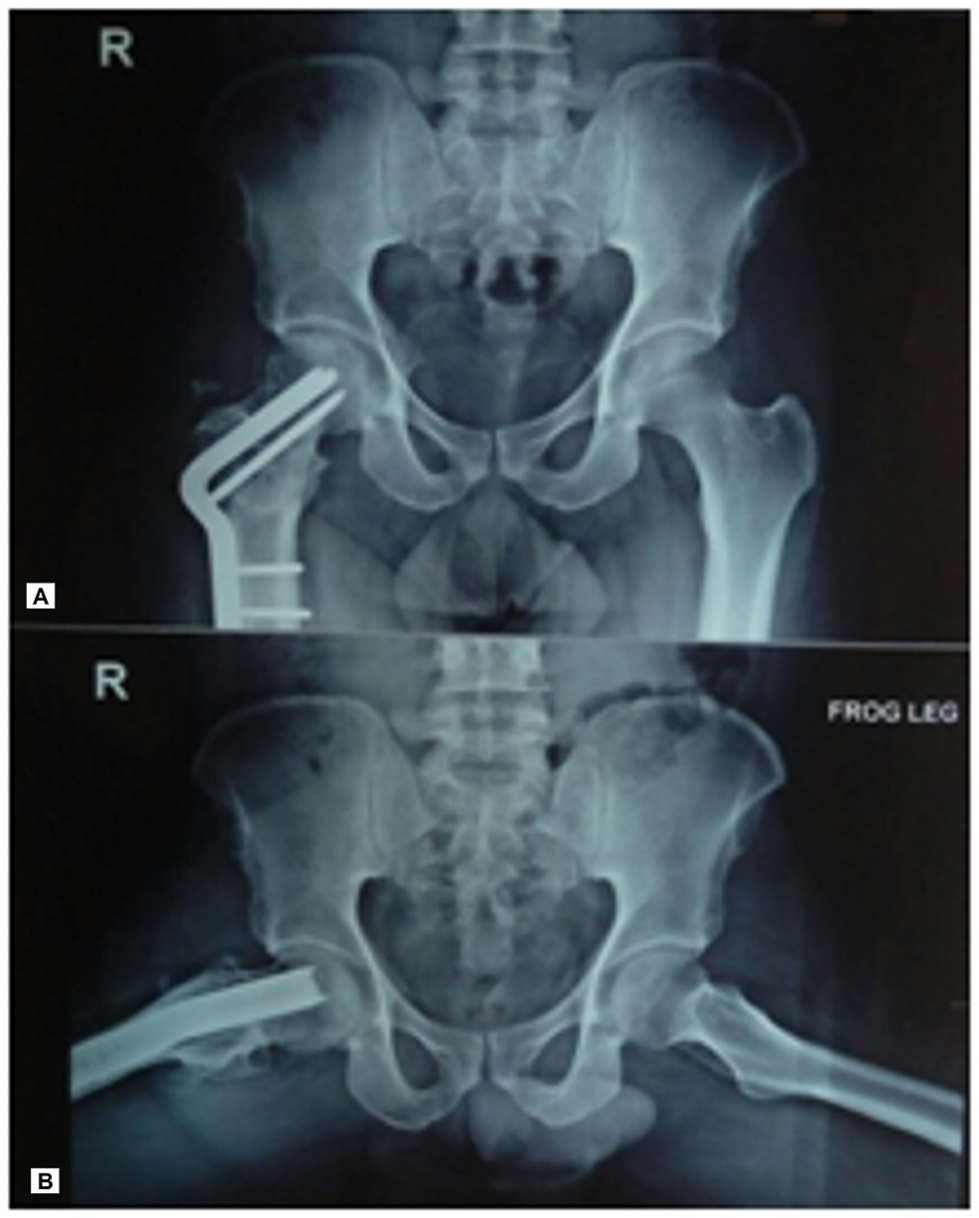
(A, B) Radiographs at four-year follow-up showing well-consolidated femoral neck with no evidence of avascular necrosis

**Figure 8:**
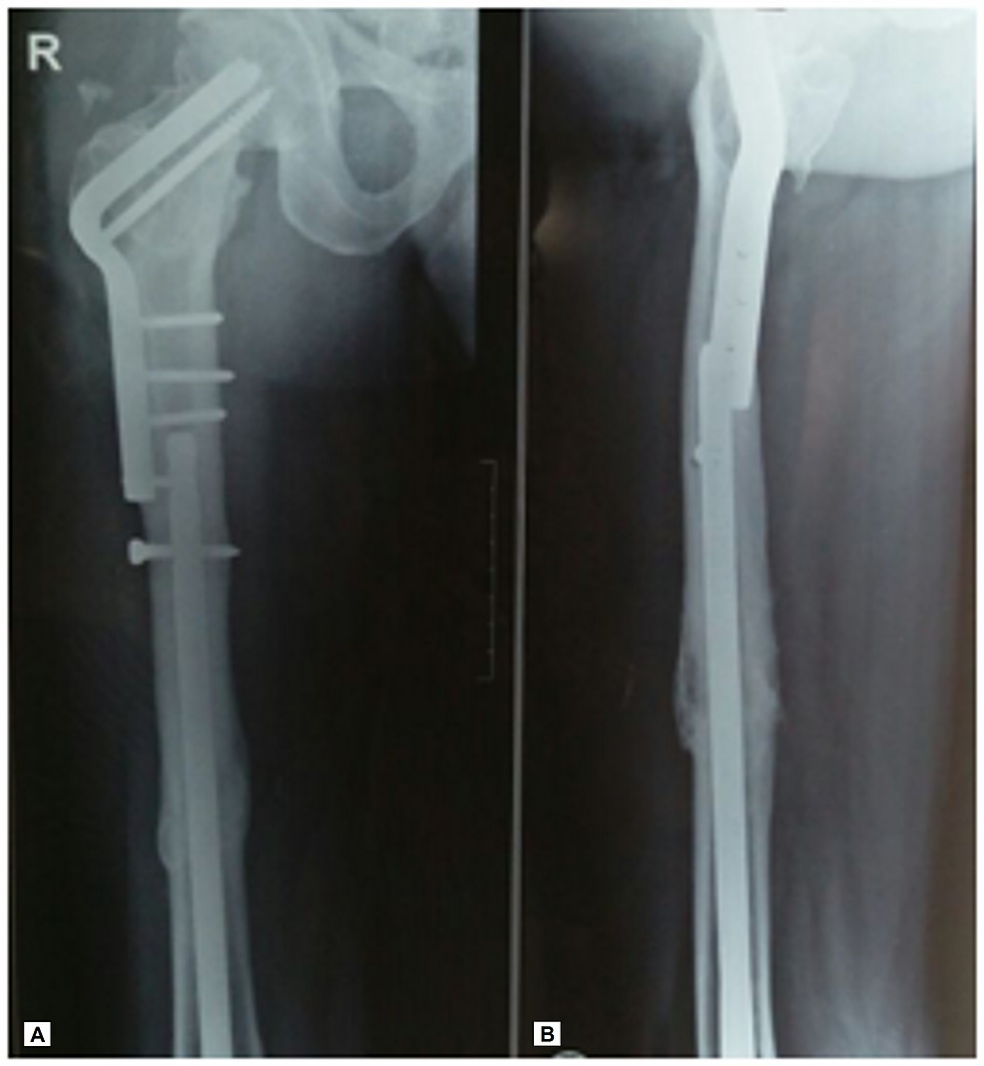
(A, B) Radiographs at four-year follow-up showing well-consolidated femoral shaft

We decided to use a retrograde nail for the shaft, without opening the fracture site, which allowed the proximal femur to be available for the Pauwels’ osteotomy. The Pauwels’ valgus intertrochanteric osteotomy acts as a biological stimulus for healing as it converts the shearing forces to compressive forces thereby promoting osteogenesis across the femoral neck nonunion site without the need to open the nonunion site. It also helps in the correction of limb shortening which is frequently seen in such presentation. While the osteotomy is relatively easy to plan and perform, often providing a definitive one-time surgery, the technique of fixation using an angled blade plate is technically challenging as it does not allow for any rotational margin of error. Angled blade plates offer the theoretical advantage of less bony resection of the femoral neck, and less iatrogenic avascular necrosis [16,17]. The use of a sliding hip screw for fixation of the valgus osteotomy has also been described [8]. The choice of the implant was especially important in view of the voids created in the femoral head and neck after removal of the pre-existing implants in our patient. Even though a sliding hip screw is a more popular and familiar implant, we were not sure how good a purchase we could get in the femoral head with a sliding hip screw. There are concerns that following the Pauwels' osteotomy, the valgus orientation of the proximal femur decreases the lever arm and therefore increases contact pressure on the femoral head which may, in turn, lead to degenerative joint disease or progression of avascular necrosis [17]. We did not note any feature of avascular necrosis in our patient at the four-year follow-up (Figures 7A, 7B and 8A, 8B).

## Conclusions

While there is a need to have increased clinical suspicion in diagnosing this injury in the acute stage, with careful planning and meticulous care, patients with concomitant nonunion of the femoral neck and shaft can be successfully treated with good outcomes. Pauwels’ valgus intertrochanteric osteotomy fixed with angled blade plate and retrograde nailing for the femoral shaft gave a satisfying outcome in our patient.
